# Biological role of the bidirectional interaction between epithelial-mesenchymal transition and PD-L1 expression in head and neck squamous cell carcinomas: A systematic review

**DOI:** 10.4317/medoral.25810

**Published:** 2023-04-07

**Authors:** Hannah Gil de Farias Morais, Carla Samily de Oliveira Costa, Rani Iani Costa Gonçalo, Leonardo Magalhães Carlan, Everton Freitas de Morais, Hebel Cavalcante Galvão, Roseana de Almeida Freitas

**Affiliations:** 1 DDS, MSc, PhD Student of Oral Pathology and Medicine, Postgraduate Program in Dental Sciences, Department of Dentistry, Federal University of Rio Grande do Norte, Natal, RN, Brazil; 2 DDS, MSc, PhD in Oral Pathology and Medicine, Postgraduate Program in Dental Sciences, Department of Dentistry, Federal University of Rio Grande do Norte, Natal, RN, Brazil; 3 DDS, MSc, PhD in Oral Pathology, Professor, Postgraduate Program in Dental Sciences, Department of Dentistry, Federal University of Rio Grande do Norte, Natal, RN, Brazil

## Abstract

**Background:**

Squamous cell carcinoma (SCC) is the most common head and neck malignant neoplasm. Despite progress in antineoplastic treatment for SCC, there are still high morbidity and mortality rates. Over the years, several tumor biomarkers have been suggested to predict the prognosis of patients with oral SCC. Studies point to a bidirectional association between the epithelial-mesenchymal transition (EMT) and the expression of PD-L1 with the aggressive biological behavior of the neoplastic cell. Thus, this systematic review aimed to explore the biological roles and mechanisms underlying the interaction between EMT and PD-L1 expression in head and neck SCC-derived cell lines.

**Material and Methods:**

An electronic search was performed in the PubMed/Medline, Web of Science, Science Direct, Scopus, Embase, and Cochrane Collaboration Library databases. Articles evaluating the *in vitro* relationship between EMT/PD-L1 interaction and the biological behavior of head and neck SCC cell lines were selected for this systematic review. The quality of evidence was assessed using the Grading of Recommendations Assessment, Development, and Evaluation (GRADE) criteria.

**Results:**

After applying the previously established inclusion/exclusion criteria, 9 articles were included in the qualitative synthesis. The present systematic review suggests the existence of a bidirectional interaction between EMT and PD-L1 expression, which is related to alterations in the cell cycle, proliferation, apoptosis, and cell survival, affecting the migration and invasion ability of tumor cells.

**Conclusions:**

Combined targeting of the two pathways may be potentially effective for immunotherapy in head and neck SCC.

** Key words:**Epithelial-mesenchymal transition, EMT, PD-L1, squamous cell carcinoma.

## Introduction

Head and neck squamous cell carcinoma (HNSCC) is the 10th most common malignancy worldwide, which is associated with high mortality rates (1-3). The treatment of HNSCC is challenging, especially in patients with advanced disease who often require a combination of different therapeutic strategies such as surgery, chemotherapy, radiotherapy and, recently, immunotherapy (1,4). The use of approaches targeting the patient’s immune system has shown promising results in cancer therapy. Thus, recent research has focused on the analysis of tumor cell extrinsic factors such as immune cells and the tumor microenvironment, relating them to immune system escape, induction of epithelium-mesenchymal transition (EMT), cell proliferation, angiogenesis, invasion, and metastasis (3,5).

EMT is a complex biological process that consists of the loss of the typical characteristics of epithelial cells, reorganization of the cytoskeleton, and the acquisition of a mesenchymal cell phenotype, which is an important step for tumor invasion and metastasis (2,5,6). Many studies have shown the association of EMT activation with metastasis, drug resistance, and consequently with a poor prognosis (5). Investigation of the molecular mechanisms underlying EMT is therefore important for understanding the processes of invasion and metastasis and for developing new therapeutic strategies (6).

The tumor microenvironment plays a crucial role in EMT. Inflammatory cytokines and immunosuppressive cells are considered key factors in EMT and distant metastasis (7). Within this context, tumor cells have developed several strategies to evade the host’s immune system, including the overexpression of programmed death-ligand 1 (PD-L1), which induces the apoptosis of immune cells by binding to programmed death-1 (PD-1) (8). Thus, most studies have associated high PD-L1 expression with a poor prognosis in different types of cancer (6).

PD-L1 (also known as CD274 or B7H1) is expressed on tumor cells and is of great importance for tumor immune escape and for the development of an immune microenvironment permissive for neoplastic growth. These processes are mediated by at least three mechanisms: (i) suppression of reactive T lymphocytes activation by PD-L1 binding to PD-1 receptor present on tumor cells surface; (ii) resistance of tumor cells to CD8+ T cells and cell lysis mediated by binding of the Fas receptor to its ligand, Fas ligand (FasL), and (iii) interaction of PD-L1 with CD80 of activated T cells, acting as an inhibitor of cell activity (9).

Several studies have shown a bidirectional association between EMT and PD-L1 expression (2,6). However, the biological mechanisms involved in this interaction have yet to be identified. Therefore, this systematic review aimed to investigate the biological roles and mechanisms underlying the interaction between EMT and PD-L1 expression in HNSCC-derived cell lines.

## Material and Methods

This meta-analysis was conducted according to the Preferred Reporting Items for Systematic Reviews and Meta-Analyses (PRISMA) guidelines (10). The study was registered with PROSPERO under number CRD42022300033. Specific questions were formulated based on the following criteria: population, intervention, control, and outcome (PICO). The research questions were “Does an interaction exist between EMT and PD-L1 expression in HNSCC?”; “What is the biological role of this interaction?”; “What are the molecular mechanisms involved in this interaction?”.

- Search strategy

To identify all primary research articles that evaluated the interaction between EMT and PD-L1 in HNSCC, we searched the following databases: PubMed/Medline, Web of Science, Science Direct, Scopus, Embase, and Cochrane Collaboration Library (last update in January 2021). A gray literature search was performed on Google Scholar, OpenGrey, and ProQuest Dissertations and Theses Global. In addition, the reference lists of the potential studies to be included in the systematic review were hand searched.

The search strategy was based on combinations of the following keywords: head and neck neoplasm, head and neck cancer, head and neck tumor, head and neck tumor, esophageal squamous cell carcinoma, oropharyngeal cancer, oropharyngeal neoplasm, oropharyngeal tumor, oropharyngeal tumor, laryngeal cancer, laryngeal neoplasm, laryngeal tumor, laryngeal tumor, oral cavity cancer, oral cavity neoplasm, oral cavity tumor, oral cavity tumor, mouth neoplasms, tongue cancer, tongue neoplasm, tongue tumor, tongue tumor, hypopharyngeal cancer, mouth neoplasms, carcinoma, squamous cell, carcinomas, squamous cell, squamous cell carcinomas, squamous cell carcinoma, carcinoma, squamous, carcinomas, squamous, squamous carcinoma, squamous carcinomas, carcinoma, epidermoid, carcinomas, epidermoid, epidermoid carcinoma, epidermoid carcinomas, carcinoma, planocellular, carcinomas, planocellular, planocellular carcinoma, planocellular carcinomas, PD-L1, programmed cell death-1 ligand-1, programmed death-ligand 1, immunotherapy, epithelial to mesenchymal transition, epithelial-to-mesenchymal transition, epithelial-mesenchymal transition, EMT, tumour microenvironment, immune system, biomarker (all fields).

- Selection criteria

Articles that assessed the relationship between the EMT/PD-L1 interaction and the biological behavior of HNSCC cell lines were selected for our systematic review. The search was conducted without time and language restrictions. The following exclusion criteria were applied: (i) studies that used only non-cancer cells; (ii) *in vivo* studies; (iii) studies that did not evaluate the interaction between EMT and PD-L1, and (iv) review articles, case reports, editorial letters, and retrospective longitudinal, cohort, case-control and randomized studies.

The articles were selected independently by three reviewers (HGFM, EFM, and LMC). Any disagreement was resolved by consensus.

- Data extraction and analysis

Five authors (HGFM, EFM, CSOC, RICG, and LMC) independently extracted the data from the included studies using a pre-established form. The extracted information included author, year, cell line, original tissue, EMT markers analyzed, induction and genetic detection methods, functional assays, signaling pathways involved, pathway direction, EMT induction (when the study evaluated the ability of EMT to induce PD-L1 expression), and main outcomes of the studies. The results of the individual studies were then summarized and the induction pathways analyzed were listed. Data for each EMT/PD-L1 direction were pooled and analyzed.

Despite differences in etiology and tumor microenvironment, given the anatomopathological similarities between HNSCC and esophageal squamous cell carcinoma (ESCC), we also included the latter in the analysis of the present study.

The quality of evidence was methodically assessed with the Grading of Recommendations Assessment, Development, and Evaluation (GRADE) tool (11,12). The GRADE tool was adapted to *in vitro* studies according to Pavan *et al*. (13), since no specific quality assessment method is available for this type of study. Five authors (HGFM, EFM, CSOC, RICG, and LMC) rated the studies as ‘high’, ‘moderate’, ‘low’, or ‘very low’ quality. If they did not reach a consensus, a fifth author (RAF) was consulted to make the final decision.

## Results

- Study selection and characteristics

**Figure 1:**
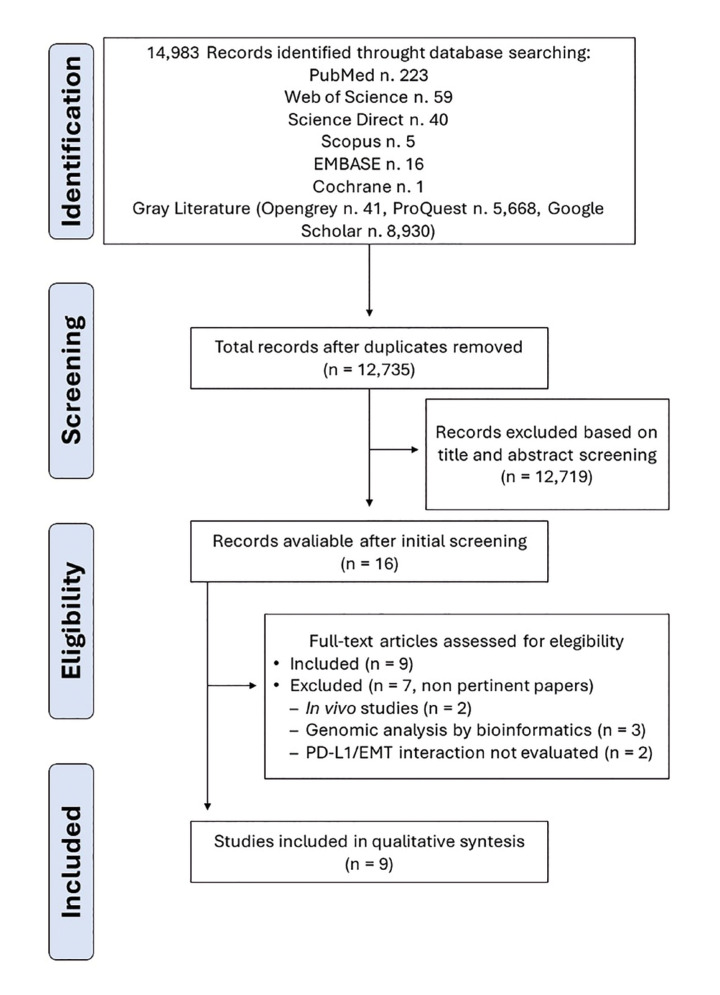
Flowchart of the article selection process according to the PRISMA guidelines.

The selection strategy developed in this systematic review retrieved 14,983 studies published in the different databases analyzed. Duplicate articles were removed using EndNote X8, resulting in 12,735 studies. After initial screening of titles and abstracts, 16 studies were considered potentially eligible and their full texts were read by five reviewers (HGFM, EFM, CSOC, RICG, and LMC). After applying the previously established inclusion/exclusion criteria, 9 articles were included (4-6,8,14-17). The PRISMA flowchart illustrates the process of article screening and selection (Fig. 1). The list of excluded studies and reasons for their exclusion are presented in Supplement 1.

The included studies evaluated 12 HNSCC and 7 ESCC cell lines with different biological behaviors and derived from different anatomical sites: esophagus (Eca-109, TE5, TE6, TE8, TE11, KYSE30, KYSE110) (1,14,15), oral cavity (OSC-19, OSC-20, TSU, PCI13, PCI8, PCI52, YD-10B, HSC-4, Cal-27) (4,5,8,17), nasopharynx (CNE2, SUNE1) (6), and hypopharynx (FaDu) (16) (Table 1).

Genetic alterations related to the induction/blockade of PD-L1 were introduced by transfection with shRNA (6) and siRNA (4,14-17). For EMT induction, the cells were treated with recombinant TGF-β1 (5,8,15) or with a GSK-3 inhibitor (1). To confirm induction, the studies analyzed the protein expression of several markers related to EMT, including epithelial (E-cadherin), mesenchymal (N-cadherin, vimentin, and TGF-β1), and transcription factors (ZEB1, Snail, and Twist1). Real-time quantitative reverse transcription polymerase chain reaction (qRT-PCR) and Western blotting were used in most of the selected studies for analysis of gene expression and quantification of protein levels, respectively.

**Table 1:**
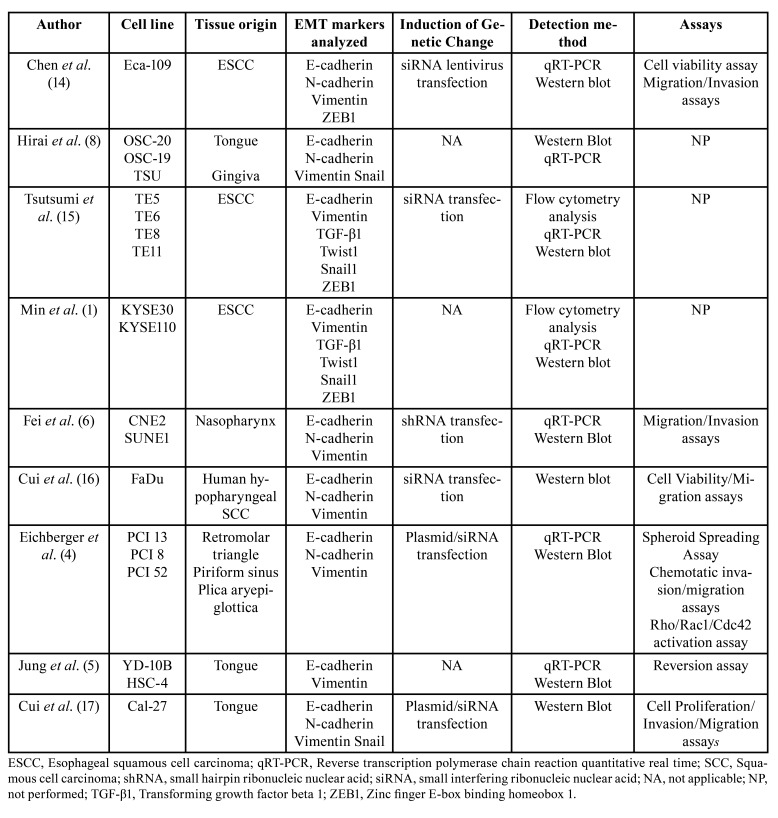
Summary of the descriptive characteristics of the included studies.

The cancer cell lines studied were submitted to functional viability, proliferation, invasion, and cell migration assays in order to elucidate the role of the interaction between PD-L1 and EMT in the biological behavior of HNSCC (Table 1). In an attempt to understand the molecular mechanisms underlying the interaction between PD-L1 and EMT in the malignant neoplasms studied, the following signaling pathways were analyzed in the present systematic review: ERK/STAT, Rho GTPase, PI3K/AKT, AKT/mTOR, and TGF-β (Table 2).

**Table 2:**
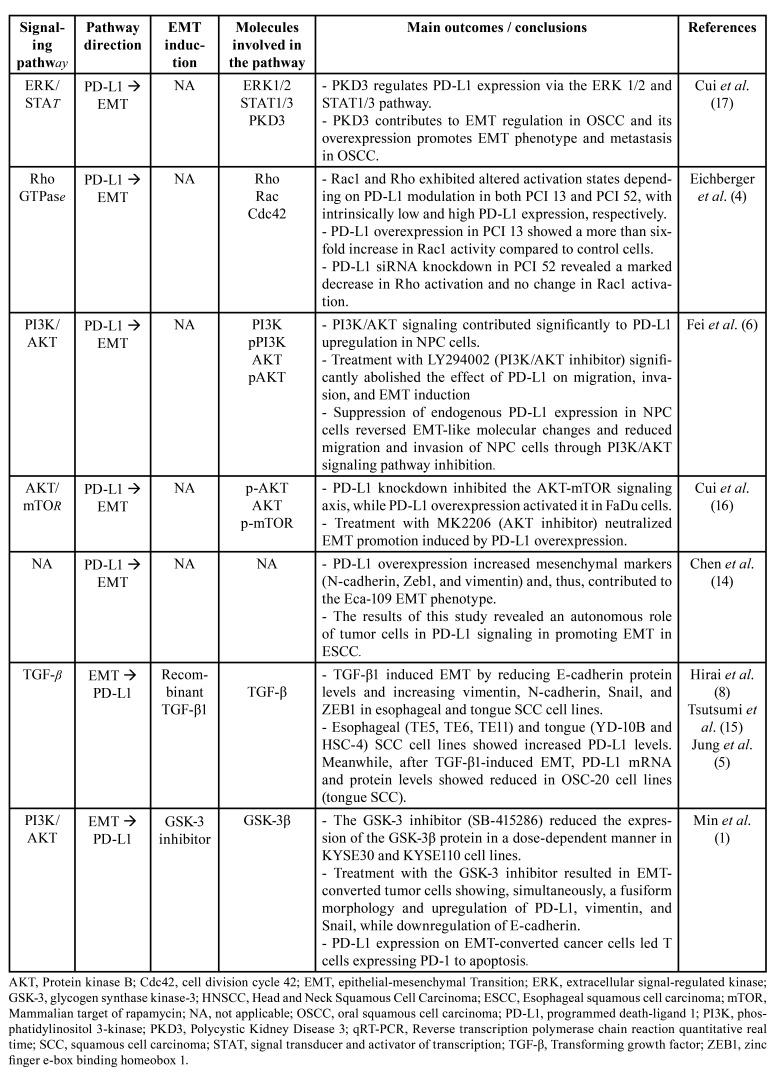
Biological role of the interaction between EMT and PD-L1 in HNSCC.

- Bidirectional interaction between EMT and PD-L1 in HNSCC cell lines

**Figure 2:**
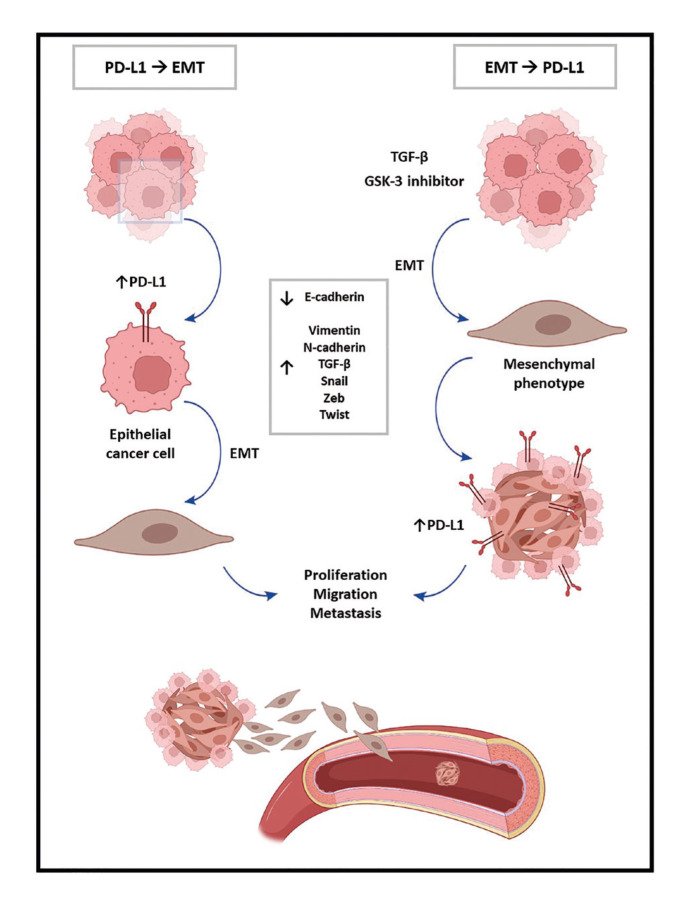
Bidirectional crosstalk of the EMT/PD-L1 interaction in HNSCC according to the results of the present systematic review. PD-L1 EMT pathway: tumor cells of epithelial origin undergo EMT triggered by PD-L1 overexpression, which induces their transformation into the mesenchymal phenotype, as well as the acquisition of a greater capacity for proliferation, migration, and metastasis. EMT PD-L1 pathway: TGF- β or GSK-3 inhibitor induces EMT in tumor cells and increased expression of PD-L1, resulting in migratory and invasive properties.

Five of the studies included in this systematic review investigated the ability of PD-L1 to induce EMT (4,6,14,16,17). In contrast, the other 4 studies analyzed the opposite direction, i.e., the ability of EMT to induce the expression of PD-L1 (Fig. 2) (1,5,8,15).

Overexpressing PD-L1 in HNSCC cell lines, Eichberger *et al*. (4) observed a reduction in E-cadherin expression levels and an increase in the levels of vimentin and of specific pluripotency regulatory markers, suggesting stimulation of EMT by PD-L1. These findings were consistent across the different cell lines used in the studies that investigated the ability of PD-L1 to induce EMT, in which PD-L1 signaling was found to play an autonomous role in the promotion of EMT in HNSCC (6,14,16,17).

Additionally, Cui *et al*. (16) demonstrated that PD-L1 knockdown in FaDu cells induced the upregulation of E-cadherin, while mesenchymal markers such as N-cadherin and vimentin were suppressed. The exact opposite results were obtained for FaDu cells in which PD-L1 was overexpressed. These findings suggest that PD-L1 blockade may act by inhibiting the EMT process in HNSCC.

In the study by Tsutsumi *et al*. (15), induction of EMT by TGF-β1 resulted in high expression of PD-L1 in ESCC cell lines. Similar results were reported by Min *et al*. (1) and Jung *et al*. (5). Min *et al*. (1) performed a co-culture experiment using EMT-converted tumor cells and IL-2 activated T cells expressing PD-1 to examine the proportion of apoptotic T cells.

The authors demonstrated that PD-L1 surface expression in these tumor cells functionally resulted in the apoptosis of PD-1-expressing T cells. Hirai *et al*. (8) reported results contrary to the previously mentioned studies, stating that invasiveness and EMT signature were inversely correlated with the expression of PD-L1 in OSC-20 cells.

- PD-L1 modulates EMT by activating the ERK/STAT, Rho GTPase, and PI3K/AKT/mTOR pathways

Cui *et al*. (17) studied the role of ERK1/2 and STAT1/3 signaling in PD-L1-induced EMT in Cal-27 cells and showed that PKD3 and PD-L1 regulate the phosphorylation levels of ERK1/2 and STAT1/3 which, in turn, can affect the expression of PKD3 and PD-L1. E-cadherin expression was increased while the expression of mesenchymal markers and PD-L1 was decreased in PKD3-knockdown cells, suggesting that PKD3 knockdown inhibits migration, invasion, and the EMT phenotype in oral squamous cell carcinoma (OSCC) cells. These data indicate that PKD3 regulates the expression of PD-L1 and EMT in OSCC through the ERK/STAT pathway, suggesting a positive feedback mechanism.

Assuming a possible role of PD-L1 as a regulator of Rho-GTPase subgroup activity, Eichberger *et al*. (4) investigated the different expression levels and activation states of the Rho family of small GTPases in OSCC lineages. qRT-PCR analysis of genes related to cell motility revealed PD-L1-dependent gene regulation associated with Rho-GTPases and genes encoding effector proteins from the Rho family of small GTPases. These findings could explain the reorganization of the neoplastic cell’s cytoskeleton during EMT, resulting in a differential behavior during tumor propagation, migration, and invasion.

In an attempt to determine whether the PI3K/AKT pathway affects EMT genes in cells that express PD-L1, Fei *et al*. (6) treated CNE2 and SUNE1 cells expressing PD-L1 with a specific PI3K/AKT inhibitor (LY294002). The results obtained for both cell lines showed significant reversal of the effect of PD-L1 on tumor migration and invasion and, consequently, on induction of the EMT phenotype. These findings suggest that PD-L1 overexpression increased the migration and invasion of NPC cells *in vitro*, inducing changes in EMT-like cellular markers through activation of the PI3K/AKT signaling pathway.

Cui *et al*. (16) examined whether PD-L1-induced EMT is regulated by the AKT/mTOR pathway and observed a significant decrease of p-Akt and p-mTOR after PD-L1 knockdown in FaDu cells. On the other hand, the expression of p-Akt and p-mTOR was significantly increased in cells transfected with the PD-L1 vector. Treatment with an AKT inhibitor (MK2206) significantly neutralized the effect of PD-L1 in promoting EMT, suggesting that PD-L1-induced EMT was indeed mediated by the AKT-mTOR pathway in FaDu cells.

- TGF-β1- and GSK-3 inhibitor-induced EMT is correlated with PD-L1 expression

TGF-β has been reported to play a role as a primary inducer of EMT in several types of cancer. Investigating whether EMT status is associated with PD-L1 expression, Jung *et al*. (5) induced EMT status in YD-10B and HSC-4 cells by treatment with recombinant TGF-β1. The authors observed that E-cadherin protein and mRNA levels were decreased in a dose-dependent manner, while vimentin levels were increased. PD-L1 mRNA and protein levels were also increased. A reversion assay was also performed to determine whether PD-L1 expression was controlled by TGF-β-induced EMT. PD-L1 mRNA and protein levels reverted to control levels after switching to the culture medium without TGF-β1. Furthermore, TGF-β1-induced upregulation of PD-L1 expression was abolished by treatment with a TGF-β1 receptor kinase inhibitor (SB 431542), suggesting that TGF-β-induced EMT status via the TGF-β signaling pathway regulates PD-L1 expression. Tsutsumi *et al*. (15) demonstrated similar results in ESCC TE5, TE6, and TE11 cell lines.

Hirai *et al*. (8) reported partially conflicting results, showing that treatment with TGF-β1 induced EMT in OSC-20 cells. Surprisingly, the total protein and mRNA levels of PD-L1 were inversely proportional to the EMT phenotype in OSC-20 cells after TGF-β1 treatment.

In contrast to other methodological approaches used in the mentioned studies, Min *et al*. (1) induced EMT in ESCC cell lines using a GSK-3 inhibitor (SB-415286), which resulted in significant downregulation of E-cadherin and upregulation of vimentin and Snail mesenchymal genes. Additionally, the authors observed morphological changes, including spindle cell morphology and a mesenchymal phenotype, as well as the loss of cell-to-cell adhesion. These results reveal that the GSK-3 inhibitor was able to induce EMT in these tumor cell lines. qRT-PCR analysis also showed an increase of PD-L1 and ZEB-1 gene expression in the GSK-3 inhibitor-treated group.

- Risk of bias in individual studies

According to the criteria established by the GRADE guidelines, the quality was low in only one study (6), moderate in four (1,15-17), and high in the other four studies (4,5,8,14) (Supplement 2).

## Discussion

EMT is a key process in tumor invasion and metastasis that allows cells to survive under diverse environmental conditions and to resist cancer treatment (5,14). In recent years, studies have demonstrated a close relationship between EMT and immune checkpoint inhibitors, especially PD-L1. This regulator has become a major focus of current research (2,7). The interaction between EMT and PD-L1 expression may induce or suppress tumor progression and survival (3). The present study demonstrated the bidirectional interaction between EMT and PD-L1 in HNSCC cell lines and its influence on mechanisms associated with progression, metastasis, tumor aggressiveness, and immune escape.

Expression of PD-L1 has been observed in tumor cells, including lung carcinoma, esophageal carcinoma, HNSCC, other types of carcinoma (colon, ovary, bladder, and breast), melanoma, and glioma (18-21). By binding to the PD-1 receptor present on immune cells, PD-L1 suppresses its effector functions and inactivates these cells, facilitating the immune escape of cancer cells (22). Thus, the PD-1/PD-L1 axis triggers immunosuppressive signals, inducing anergy of cytotoxic T cells, which has been closely related to a poor prognosis (6).

The present systematic review analyzed the existence of bidirectional crosstalk between EMT and PD-L1 in different HNSCC cell lines. PD-L1 is essential not only for immunosuppression but also for EMT, playing an important role in tumorigenesis where it is associated with reduced cell-to-cell adhesion, gain of the mesenchymal phenotype, and increased rates of invasion/migration (6,14,16,17). These findings provide a strong rationale for blocking the PD-1/PD-L1 axis by immuno-oncology (23), especially in the case of aggressive tumors with high recurrence and relapse rates whose treatment is challenging, such as HNSCC (1). Randomized controlled trials have shown promising results in the treatment of these tumors with the anti-PD-1 monoclonal antibodies pembrolizumab and nivolumab (24,25). Ferris *et al*. (24) compared the efficacy of nivolumab to that of single-agent chemotherapy in 347 patients with HNSCC and observed significant improvement in survival in the nivolumab group. Likewise, a phase III study conducted by Cohen *et al*. (25) compared the clinical efficacy of pembrolizumab versus current standard therapy in 495 patients and found significant improvement in survival in the pembrolizumab group. Thus, immunological monitoring of patients is a valuable tool to identify potential biomarkers and to stratify and accurately delineate responders and non-responders in order to optimize the immunostimulatory effects of therapeutic agents.

Changing the direction of the pathway, in concern to EMT-induced PD-L1 expression, the results of this systematic review are controversial. Tsutsumi *et al*. (15), Min *et al*. (1), and Jung *et al*. (5) showed that induction of EMT by TGF-β1 resulted in high expression of PD-L1. Furthermore, Min *et al*. (1) demonstrated that the expression of PD-L1 in EMT-induced ESCC cells triggered apoptosis of T cells expressing PD-1. In contrast, Hirai *et al*. (8) found that invasiveness and EMT signature were inversely correlated with the expression of PD-L1 in OSC-20 cells; thus, the neoplastic cells maintained their high-grade invasiveness even after downregulation of PD -L1. The most likely explanation for this paradox is that PD-L1 expression is regulated, among other mechanisms, by cytokines, especially IFN-γ present in the tumor microenvironment. IFN-γ expression would thus reflect the contribution of the endogenous antitumor immune response, which typically occurs in the early stages of tumor development and progression (26). This review showed that the EMT process can induce cancer growth and metastasis through the action of immune checkpoint molecules that reprogram immune activity in the tumor microenvironment.

EMT-inducing transcription factors are generally activated by different signaling pathways, with the TGF-β-mediated pathway being the most widely accepted mechanism for the induction of EMT (27,28). Tsutsumi *et al*. (15) showed that TGF-β1-induced EMT resulted in high expression of PD-L1 in ESCC cell lines, similar to the findings of Jung *et al*. (5). TGF-β1 exerts diverse effects in cancer, supporting tumor progression by favoring metastasis and inhibiting antitumor immunity (29). Similar results have been reported by Min *et al*. (1) who induced the EMT phenotype by inhibiting GSK-3, which is located at the end of the PI3K-AKT signaling pathway. Thus, the TGF-β1 and PI3K-AKT pathways could be useful therapeutic targets since downregulation of these pathways may negatively interfere with EMT/PD-L1 crosstalk.

However, other pathways influence the expression of PD-L1 and EMT markers, a fact that renders the elucidation and determination of the check points of these pathways a challenge. Cui *et al*. (17) demonstrated that PKD3 regulates the expression of PD-L1 and EMT proteins in OSCC through the ERK/STAT pathway, suggesting a positive feedback mechanism. Fei *et al*. (6) found that PI3K/AKT signaling contributed significantly to the upregulation of PD-L1 in CNE2 and SUNE1 cells and the induction of the EMT phenotype. Eichberger *et al*. (4) suggested the Rho-GTPase pathway as a possible regulator of PD-L1 in OSCC lineages. According to Cui *et al*. (16), the AKT-mTOR pathway mediates PD-L1-induced EMT in FaDu cells.

A challenge in regulatory science is the identification of appropriate biomarkers that could lead to the approval of immunotherapeutic approaches for each tumor conFiguration (3). Low immunotherapy response rates in metastatic and/or recurrent HNSCC indicate a lack of understanding of the immunobiology of these diseases, as well as of the mechanisms involved in the therapeutic response and resistance (30). Thus, this systematic review proposes that the co-inhibition of PD-L1 expression and EMT status through the TGF-β and PI3K-AKT pathways may provide new insights into the inhibition of tumor invasion and metastatic progression in these malignancies. Therefore, future prospective studies associating immunological checkpoint and EMT biomarkers and, in addition, the development of *in vitro* diagnostic tests are necessary, always considering the neoplastic and patient heterogeneity.

## Conclusions

In summary, the present systematic review demonstrated the existence of a bidirectional interaction between EMT and PD-L1 expression in HNSCC-derived cell lines. We also showed that this interaction is regulated by several signaling pathways related to the cell cycle, proliferation, apoptosis, and cell survival, interfering with the spread of neoplastic cells and tumor survival. These findings suggest the existence of a cooperative mechanism between tumor imune microenvironment and EMT, which may provide more information about the possible crosstalk between EMT genes and antitumor imune response in HNSCC. More importantly, this interaction indicates that combined targeting of the two pathways may be more effective for current immunotherapy. However, further *in vitro* and *in vivo* studies are necessary to elucidate the exact molecular mechanisms underlying the association between EMT and the regulation of PD-L1 expression in the tumor microenvironment.
